# Breakfast in the United States: Food and Nutrient Intakes in Relation to Diet Quality in National Health and Examination Survey 2011–2014. A Study from the International Breakfast Research Initiative

**DOI:** 10.3390/nu10091200

**Published:** 2018-09-01

**Authors:** Adam Drewnowski, Colin D. Rehm, Florent Vieux

**Affiliations:** 1 Center for Public Health Nutrition, University of Washington, Box 353410, Seattle, WA 98195, USA; 2 Albert Einstein College of Medicine, Montefiore Medical Center, New York, NY 10467, USA; colin.rehm@gmail.com; 3 MS-Nutrition, 27 bld Jean Moulin Faculté de Médecine la Timone, Laboratoire C2VN, CEDEX 5, 13385 Marseille, France; florent.vieux@ms-nutrition.com

**Keywords:** breakfast, dietary intake, nutrition, dietary quality, NRF9.3 index, USDA HEI 2015 index

## Abstract

The contribution of breakfast to diet quality (DQ) can inform future dietary guidelines. This study examined breakfast nutrition in relation to overall DQ, using dietary data from the first reported day of the National Health and Examination Survey (NHANES) 2011–2014 (*n* = 14,488). Relative DQ was assessed using the Nutrient Rich Foods Index (NRF9.3) and the USDA Healthy Eating Index 2015 (HEI 2015). The sample was stratified by NRF9.3 tertiles and by age and socioeconomic groups. Four out of 5 NHANES participants had breakfast on the day of the interview. Breakfast provided 19–22% of dietary energy depending on age. Breakfast intakes of complex carbohydrates and total sugars were proportionately higher and intakes of protein and fats were lower relative to breakfast energy intakes. Breakfast provided more that 20% of daily intakes of B vitamins, vitamins A and D, folate, calcium, iron, potassium and magnesium. Eating breakfast was associated with higher NRF9.3 DQ scores. Breakfasts associated with the top tertile of NRF9.3 scores had less added sugars and fats than those associated with the bottom tertile. Such breakfasts had more fruit and juices, more whole grain products, more milk and yogurt and less meat and eggs. Breakfast patterns and food choices that favored fruit, whole grains and dairy were associated with healthiest diets.

## 1. Introduction

Breakfasts that provide more nutrients than calories can be viewed as nutrient-rich meals [1,2,3,4]. Eating breakfast has been associated with higher-quality diets and with higher intakes of key nutrients and desirable food groups [5,6]. By contrast, skipping breakfast has been linked to lower-quality diets, lower cognitive performance and a host of negative health outcomes [7,8,9,10,11,12,13,14,15]. The International Breakfast Research Initiative (IBRI) aimed to identify breakfast patterns associated with highest quality diets using nationally representative data from six countries: Canada, Denmark, France, Spain, UK and the US.

Analyses of NHANES 2001–2008 data showed that about 19% of the US population skipped breakfast altogether [4]. The rest exhibited as many as 12 breakfast “patterns” that typically included grain products, fruit juice, milk, whole fruit, sweets, meat and eggs and coffee or tea [4]. In some studies, the consumption of selected breakfast components (e.g., ready to eat or RTE cereals) was associated with higher-quality diets [16,17,18,19]. What food groups make for a healthy breakfast pattern across countries and consumer subgroups continues to be a topic of research interest [1,5,20,21].

This study examined the notion that the US breakfast is a nutrient-rich meal by assessing the contribution of breakfast to daily energy and nutrient intakes among US children and adults. Breakfast patterns associated with different-quality diets were then examined in detail. The goal was to arrive at an optimal combination of breakfast foods that could be the basis of future dietary recommendations and guidelines.

## 2. Materials and Methods

### 2.1. Study Population & Dietary Data

Analyses were based on the first day of dietary intakes in the 2011–2012 and 2013–2014 cycles of the nationally representative National Health and Nutrition Examination Survey (NHANES) [22,23]. Data were available for 14,488 children, adolescents and adults aged ≥ 6 y. The sample included 2511 children (ages 6–12 y); 1546 adolescents (ages 13–17 y); 6594 adults (ages 18–54 y) and 3837 older adults (ages ≥ 55 y).

The first 24-h recall in the NHANES was completed in-person at the Mobile Examination Center with a trained interviewer. The 24-h recall queries all foods/beverages consumed by participants from midnight-to-midnight on the previous day [22,23]. Dietary supplements were excluded. Breakfast was defined as the self-reported “breakfast/desayuno” and brunch. An energy threshold of 50 kcal was imposed. Breakfast skippers were defined as having no breakfast or an eating episode of <50 kcal.

The population sample was stratified by four age groups (6–12 y, 13–17 y, 18–54 y and ≥55 y) and six race/ethnicity groups (non-Hispanic white, non-Hispanic black, Mexican-American, other Hispanic, Asian and other/mixed race). Education was defined as: <High School (<12 y), High School (12 y); Some college (12–16 y) and >College (>16 y). Income to poverty ratio (IPR) cut-points were set at: <1.3; 1.3–1.849; 1.85–2.99; >3.

### 2.2. Measures of Diet Quality

The Nutrient Rich Foods (NRF) index was the principal measure of nutrient density of the total diet [19,24,25]. Its development and validation, with respect to other measures of diet quality and long-term health outcomes, have been described in the literature [24,25,26,27]. The present NRF9.3 variant applied to total diets was based on 9 qualifying nutrients (NR) and 3 disqualifying nutrients (LIM). Reference daily values (DVs) were based on the US Food and Drug Administration (FDA) and other standards [19,24]. The qualifying nutrients and standard reference amounts were as follows: protein (50 g), fiber (28 g), vitamin A (900 RAE), vitamin C (90 mg), vitamin D (20 mcg), calcium (1300 mg), iron (18 mg), potassium (4700 mg) and magnesium (420 mg). The 3 disqualifying nutrients and maximum recommended values (MRVs) were: added sugar (50 g), saturated fat (20 g) and sodium (2300 mg). The NRF9.3 was calculated as follows:

NRF9.3 = (NR − LIM) × 100(1)

with

(2) NR=∑i=19IntakeiEnergy×2000DVi

and

(3) LIM=∑i=13IntakeiEnergy×2000MRVi−1

where *intake_i_* is the intake of each nutrient *i* and *DV_i_* is the reference daily value for that nutrient.

In NR calculation, each daily nutrient intake *i* was adjusted for 2000 kcal and expressed in percentage of DV. Following past protocol, percent DVs for nutrients were truncated at 100, so that an excessively high intake of one nutrient could not compensate for the dietary inadequacy of another. In LIM, only the share in excess of the recommended amount was considered.

The development and validation of the NRF family of nutrient density scores are all well-documented in the literature [26,27]. In the present adaptation, vitamin D, a nutrient of public health concern [28,29,30], replaced vitamin E. Fiber, vitamin D, calcium, magnesium and potassium were all identified in the 2010 Dietary Guidelines for Americans as nutrients of concern [29]. The NRF score was adjusted for energy intakes, analogous with the recent versions of the USDA Healthy Eating Index (HEI), a federal measure of diet quality [31].

The HEI-2015 is the latest iteration of the USDA diet quality measurement tool, specifically designed to monitor compliance with the 2015 Dietary Guidelines for Americans [31]. The HEI-2015 is a 100-point scale where the adequacy components are total fruits (5 points), whole fruits (5), total vegetables (5), greens and beans (5), whole grains (5), dairy (10), total protein (5), seafood and plant protein (5) and fatty acid ratio (10); the moderation components are refined grains (10), sodium (10) added sugars (10) and saturated fats (10). HEI 2015 values were calculated using the USDA Food Patterns Equivalents Database (FPED) [32]. Both NRF9.3 and HEI 2015 were corrected for dietary energy (1000 kcal for HEI and 2000 kcal for NRF).

### 2.3. Analytical Strategy

Energy and nutrient intakes for NHANES participants were calculated using the Food and Nutrient Database for Dietary Studies 2011–2014. The primary nutrient outcome measures were selected based on their overall importance to current dietary recommendations [29]. Some of the nutrients were in the NRF model but some were not. For example, fiber, vitamin D, calcium, magnesium and potassium (all in the NRF model) were identified in the 2010 Dietary Guidelines for Americans as nutrients of concern [29]. Iron (also in the model) was identified as a nutrient of concern for adolescent girls and women capable of becoming pregnant. By contrast, the NRF model did not include nutrients of concern such as folic acid (women capable of becoming pregnant) or vitamin B12 (older adults) [19,24]. Breakfast food groups of interest were based on reported consumption frequency by children and adults and included milk, whole fruit and fruit juices, whole grains and low-fat dairy, soy, nuts and legumes, as well as ready to eat cereals (RTEC).

All analyses were conducted using SAS software, Version 9.4 (SAS Institute Inc., Cary, NC, USA) and are representative of the US population. Differences between proportions were tested using X² tests. Differences in quantitative variables (such as intakes) were tested using Generalized Linear Models, adjusted as appropriate (without and with adjustment for energy at breakfast as well as energy at breakfast and socio-demographics characteristics). Pearson coefficient correlations between NRF9.3 and HEI score, as well as between NRF9.3 and all HEI subscores, were estimated. The statistical significance level was set at *p*-value < 0.05.

### 2.4. Data Availability and Ethical Approval

The necessary Institutional Review Board (IRB) approval for NHANES had been obtained by the National Center for Health Statistics (NCHS) [33]. Adult participants provided written informed consent. Parental/guardian written informed consent was obtained for children. Children/adolescents ≥ 12 y provided additional written consent. All NHANES data are publicly available on the NCHS and USDA websites [22,23]. Per University of Washington (UW) policies, public data do not involve “human subjects” and their use requires neither IRB review nor an exempt determination. Such data may be used without any involvement of the Human Subjects Division or the UW Institutional Review Board.

## 3. Results

Table 1 shows that out of 4057 children, 3296 (82.0%) ate breakfast on the first day of the NHANES survey and 761 did not (18.0%). Out of 10,431 adults, 8269 (80.3%) ate breakfast and 2162 (19.7%) did not. Those figures were based on the <50 kcal breakfast energy threshold. With the threshold removed, 17.4% of children and 15.2% of adults ate no breakfast at all.

Breakfast consumption patterns showed a bimodal distribution by age. Most likely to eat breakfast (87.5%) were young children and older adults. Only 3 out of 4 adolescents and young adults ate breakfast. Among children, most likely to eat breakfast were Asians, Whites and other Hispanics. Least likely to eat breakfast were non-Hispanic Blacks. Among adults, most likely to eat breakfast were non-Hispanic Whites, other Hispanics and Asians. Least likely to eat breakfast were non-Hispanic Blacks. Breakfast consumption increased sharply with household incomes for children and adults and with education and incomes for adults. Higher-income groups and college graduates were most likely to eat breakfast.

**Figure 1:**
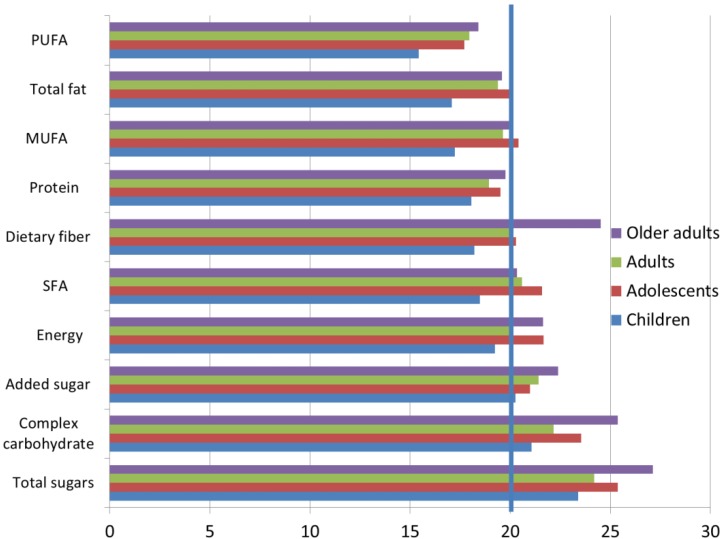
Percent contribution of breakfast to macronutrient intakes relative to energy intakes among breakfast consumers. PUFA stands for Polyunsaturated fatty acids, MUFA stands for Monounsaturated fatty acids, SFA stands for Saturated fatty acids. The 20% cutoff is indicated by a vertical line.

Subsequent analyses were conducted among breakfast consumers only. Figure 1 shows the percent contribution of breakfast to total daily energy and nutrient intakes by age group. For the whole NHANES sample, mean and median energy intakes at breakfast were 447 kcal/d and 366 kcal/d, respectively. Breakfast accounted for approximately 20% of daily energy intakes. The exact percentages were 19.2% of energy intakes for children, 21.7% for adolescents; 20.0% for adults and 21.8% for older adults.

Breakfast supplied just <20% of daily protein and total fat, approximately 20% of fiber and saturated fatty acids (SFA) and around 25% of total sugars and between 20% and 22% of added sugar, depending on age. Older adults consumed more dietary fiber, carbohydrates and total and added sugars at breakfast than did children and adolescents.

**Figure 2:**
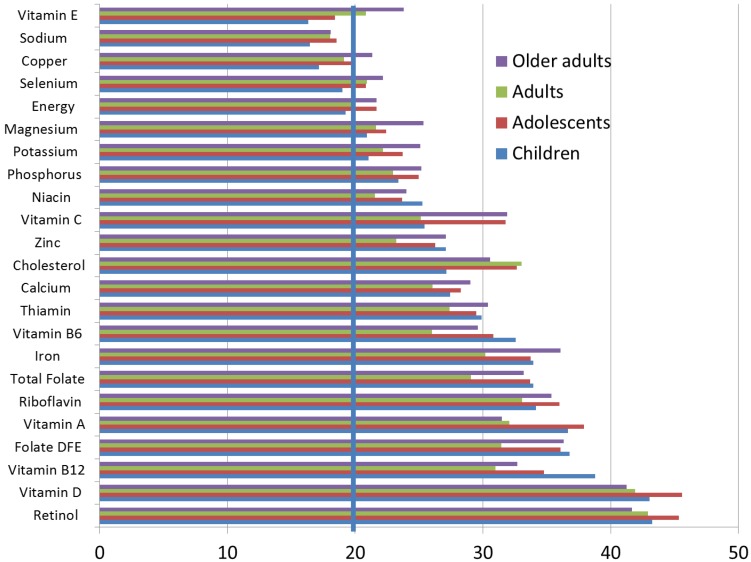
Percent contribution of breakfast to micronutrient intakes relative to energy intakes among breakfast consumers. The 20% cut point is indicated by the vertical line.

Figure 2 shows percent contribution of breakfast to total dietary energy and micronutrient intakes by age group. Although the energy contribution was about 20%, breakfast provided substantially more than 20% of daily magnesium, potassium, phosphorus, niacin, vitamin C, zinc, calcium, thiamin, vitamin B6, iron, folate, riboflavin, vitamin A, folate, vitamin B12, vitamin D and retinol. For all age groups, breakfast provided >40% of daily vitamin D. While the percentage of sodium was <20%, the percentage of cholesterol from breakfast was in the order of 30%.

### 3.1. Measures of Diet Quality—NRF9.3

**Figure 3:**
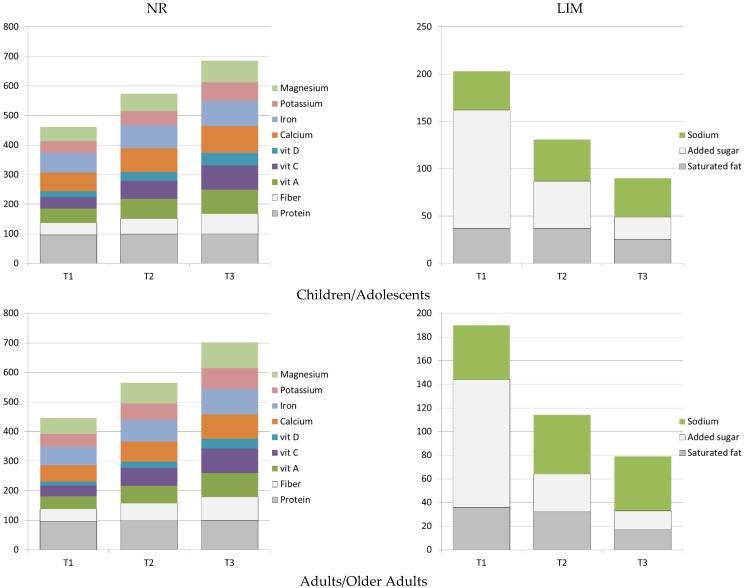
Nutrient subscores of the Nutrient Rich Index (NRF9.3) by age group and by tertiles of total NRF9.3 scores.

Figure 3 summarizes the construction of the NRF9.3 score, used here as a measure of nutrient density of the total diet. The NRF9.3 was adjusted per 2000 kcal, as detailed above. Separate panels show the NR subscore, composed of nutrients to encourage and the LIM subscore, composed of nutrients to limit. Figure 3 shows that percent daily values for index nutrients rose with tertiles of the NRF9.3 score, whereas the LIM subscores, to the contrary, decreased. As expected, going from the lowest (T1) to the highest tertile (T3) of NRF9.3 scores was associated with an increase in percent DVs of nutrients to encourage and a corresponding decrease in percent MRVs of nutrient to limit [24,27].

The correlation between NRF9.3 scores and HEI 2015 scores based on the entire population aged >2 was statistically significant, *r* = 0.43. The correlation between NRF9.3 scores and HEI subscores was statistically significant for most HEI components (*r* = 0.2 to *r* = 0.34) and was strongest for added sugars, dairy, whole fruit and total fruit. Previous studies have shown that HEI scores were sensitive to age, gender and sociodemographic characteristics of NHANES study participants.

Table 2 shows mean DQ NRF9.3 scores for NHANES breakfast consumers by age, gender and sociodemographic characteristics. First, there was a bimodal effect of age—highest quality diets were consumed by children and by older adults; by contrast, adolescents had lowest-quality diets, consistent with many other reports [34,35]. Gender effects depended on age; whereas no gender differences were observed for children or adolescents, adult women had more nutrient-dense diets than did men.

The most nutrient-dense diets were consumed by Asians and other Hispanics. Non-Hispanic Blacks had lowest quality diets at every age. Diet quality of adults greatly improved with education and with household incomes. An income gradient for children was not observed. Differences in NRF scores by education and incomes were far greater than those observed by race/ethnicity.

Skipping breakfast had profound effects on NRF9.3 scores in univariate analyses. For children the difference was 107 points (Consumers = 449; skippers = 342); for adolescents, the difference was 80 points (Consumers = 407; skippers = 327); for adults, it was 110 points (Consumers = 420; skippers = 310) and for older adults the difference was 95 points (Consumers = 483; skippers = 388).

### 3.2. Breakfast Patterns by Tertiles of NRF9.3 Diet Quality Scores

**Figure 4:**
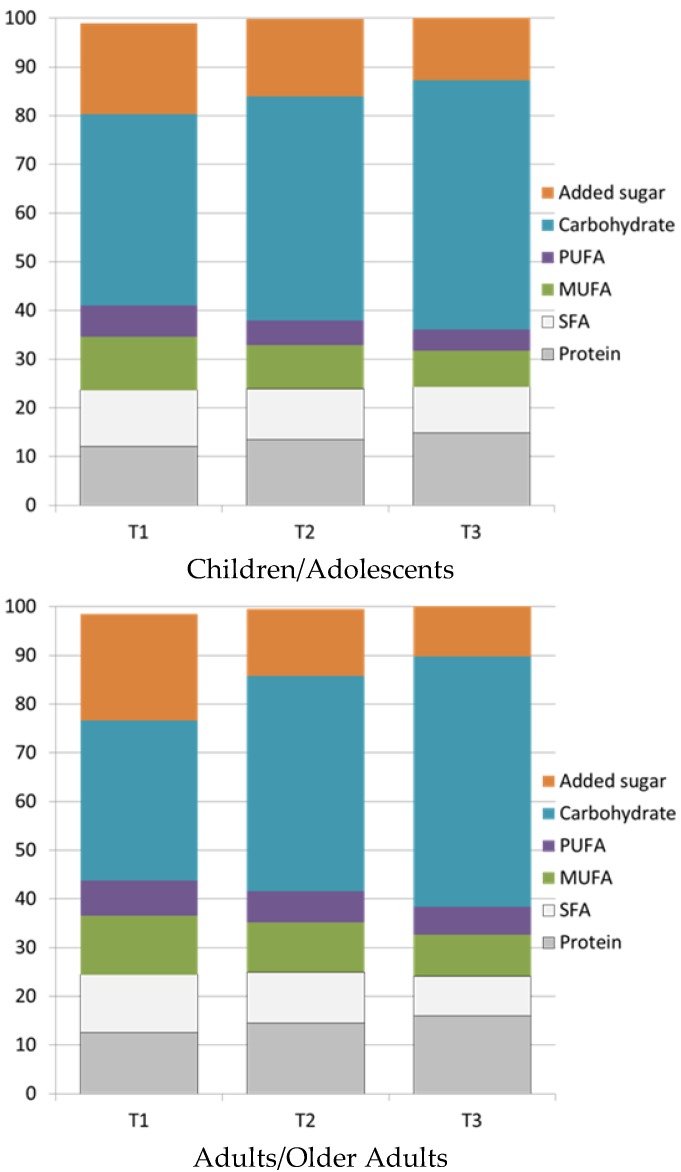
Distribution of breakfast macronutrients by tertiles of NRF9.3 diet quality score. PUFA stands for Polyunsaturated fatty acids, MUFA stands for Monounsaturated fatty acids, SFA stands for Saturated fatty acids.

Figure 4 shows the macronutrient composition of breakfasts associated with tertiles of NRF9.3 scores. Breakfasts associated with better diets had much less added sugar and less fat but more carbohydrate and slightly more protein.

The amounts of specific food groups consumed by diet quality tertiles are shown in Table 3, separately for children and for adults. First, higher-quality diets were associated with higher consumption of citrus fruit, juice and other fruits, whole grains and milk and yogurt. The consumption of citrus fruit, juice and other fruits doubled or tripled. The consumption of refined grains was cut in half but the consumption of whole grains almost tripled. Higher-quality diets were associated with lower consumption of refined grains, breakfast meats, eggs and cheese. Meat, poultry and seafood were substantially reduced; there was an increase in consumption of soy, nuts and legumes. The consumption of milk and yogurt increased, cheese dropped slightly. Solid fats were sharply reduced. Among adults, higher quality diets were associated with higher breakfast consumption of soy, nuts and legumes.

**Figure 5:**
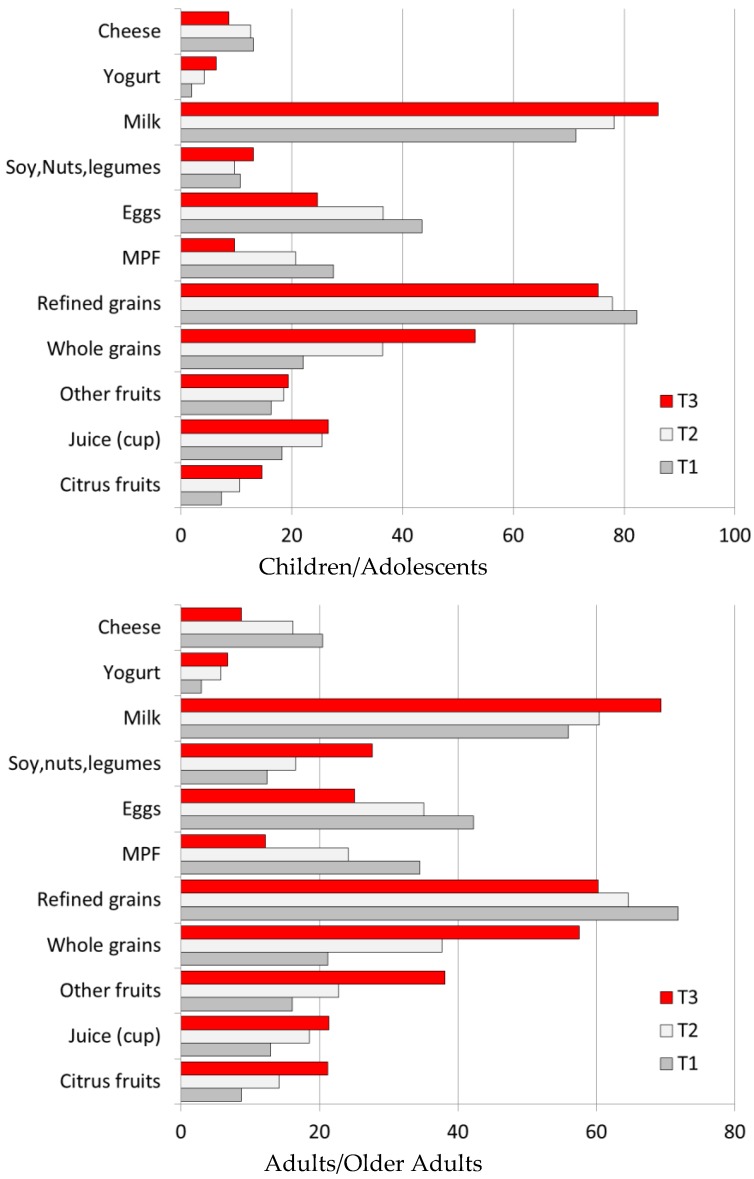
Percent consumers at breakfast for selected food groups by NRF9.3 tertiles. MPF stands for Meat, Poultry, Fish.

Percentages of breakfast consumers of specific food groups by diet quality tertiles are shown in Figure 5. First, higher quality diets were associated with more children and adults consuming citrus fruit, juice and other fruits, whole grains and milk and yogurt at breakfast. Higher quality diets were associated with fewer people consuming refined grains, breakfast meats, eggs and cheese. Higher quality diets were associated with more adults consuming soy, nuts and legumes.

Table 4 shows the association between breakfast micronutrients and tertiles of the NRF9.3 score. As expected, there was an increase in the intake of nutrients that were in the model (protein, fiber etc.). There was also an increase in the intake of qualifying and shortfall nutrients that were not in the model. The latter include B vitamins, B12, folate and others.

**Figure 6:**
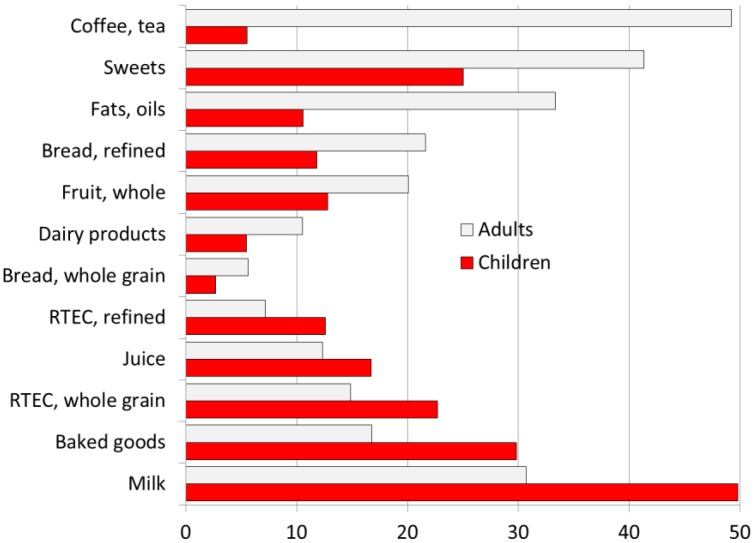
Percent consumers of specific food groups at breakfast by age group (data for breakfast consumers only). RTEC stands for Ready to eat cereals.

Lastly, Figure 6 shows the most common food eaten at breakfast by US children and adults. For children, the most frequently consumed foods were milk, baked goods, sweets, whole grain RTEC, juice and whole fruit. For adults, the most frequently consumed items were coffee or tea, sweets (including sugar), baked goods, fats, white bread and whole fruit. The consumption of low fat dairy and whole grain bread was low.

## 4. Discussion

The present analyses of first-day data from the 2011–2014 NHANES data asked what breakfast food patterns were associated with most nutrient-rich diets [1,4,6,19,36]? Shifting the focus from individual nutrients to food choices and food patterns can pave the way for future food-based dietary recommendations and guidelines. The 2015 Dietary Guidelines Advisory Committee has already delineated the relation between food patterns and health outcomes [29]; the emphasis on healthy food choices and healthy food patterns is likely to continue.

First, breakfast consumption contributed to overall DQ. Four out of 5 of NHANES participants in the 2011–2014 database ate breakfast on the first day of dietary data collection. Breakfast consumption was associated with higher socioeconomic status and also with higher-quality diets. NRF9.3 scores were higher for breakfast consumers than for non-consumers for every age group. Breakfast skipping was associated with lower education and incomes, themselves predictors of lower-quality diets and impaired health [37]. Many previous studies have pointed to associations between breakfast skipping and unfavorable health outcomes [13,14,38,39,40,41,42].

Among breakfast consumers, breakfast provided about 20% of daily energy, depending on age. Many micronutrients, vitamins and minerals were provided in amounts exceeding 20% of daily intakes. The definition of nutrient density in the 2005 Dietary Guidelines [43] defined nutrient dense foods as those containing “more nutrients than calories.” Based on a simple nutrients-to-energy ratio, breakfast can be considered a nutrient rich meal. Depending on age, breakfast provided >20% of daily carbohydrate and total sugar and approximately 20% of protein and fats.

The Nutrient Rich Foods Index (NRF9.3), adapted for use with total diets was the principal measure of DQ. As expected, DQ improved with age. Consistent with past studies, NRF9.3 scores were associated with higher education and incomes. Asians had the highest NRF9.3 scores; non-Hispanic Blacks had the lowest. In past studies, HEI 2010 scores were likewise associated with higher SES.

Diets that scored in the top NRF9.3 tertile were associated with higher intakes of some key nutrients, including those that were in the model and some that were not. Diets in the top NRF9.3 tertile were also associated with higher consumption of some desirable food groups and with a higher prevalence of their consumption. The present conclusion is that the NRF9.3 nutrient density score, initially developed to capture nutrient density of individual foods, also captured nutrient density of the total diet. As expected, higher NRF scores were associated with higher SES. The SES gradient in diet quality was significant for adults but not for children and teenagers.

Even though the typical US breakfast breakfasts provided more nutrients than calories, there was room for improvement in breakfast quality. For children, the typical breakfast foods were milk, baked goods and sweets. Whole grain RTEC and whole fruit further down on the list. Adult breakfast foods included coffee/tea, sweets, fats and white bread. The present analyses allowed us to identify those food choices and breakfast patterns that were associated with highest quality diets, as captured by NRF9.3 scores. Among adults, those optimal patterns were characterized by higher intakes of citrus fruit, whole fruit and juice, soy, nuts and legumes. Among children, the optimal breakfast patterns were characterized by higher intakes of whole grain cereals, more milk and yogurt and by lower intakes of animal protein, less meat, eggs and saturated fats.

The food- and nutrient-based approach to the optimum breakfast is consistent with federal guidelines. The current federal guidelines for nutrition standards in the national school lunch and school breakfast program are both food and nutrient based. Their goal was to provide nutrient rich meals (high in nutrients and low in calories) to meet the dietary needs of schoolchildren. Rules to reduce sodium, saturated fat and trans fat were accompanied by rules to increase the availability of fruits, vegetables, whole grains and skim and low-fat milk on the school menus [44].

The present results, showing that the optimum breakfast was higher in fruit and juice, whole grain cereals and milk and yogurt, have implications for future dietary recommendations and guidelines. The IBRI dietary analyses deliberately focused on breakfast food groups and food categories in addition to individual nutrients. The 2020 Dietary Guidelines for Americans will most likely continue to focus on food patterns, showing how culturally different food patterns can result in nutrient rich meals. Nutrient profiling will most likely follow suit. Whereas most existing nutrient density scores are based on nutrients alone [27], future nutrient profiling models may take a hybrid approach, one that includes desirable foods or food groups alongside nutrients to encourage and nutrients to limit. Such desirable food groups may include fruits, nuts, seeds, whole grains and low-fat dairy. The notion of what constitutes a healthy food is being revisited by the US Food and Drug Administration.

The limitations of this study are worth noting. First, all population based dietary data in the US and the 5 other countries were based on self-report. While self-reports may not reflect true dietary intakes, the fact is that most representative population based dietary intake data globally are based on self-report. Second, data analyses were based on the first day of the 2-day NHANES survey. One day recalls are a reliable way to assess nutrient intakes of populations but do not capture the habitual dietary patterns of the individual. Better able to address habitual dietary patterns are the national dietary surveys in France, based on 7-day diaries and those in the UK, based on 4 days. Third, the breakfast meal was defined by self-report (breakfast or brunch) as opposed to the time of day. In some past studies, the timing of the meal served to define breakfast. Fourth, the food groups of interest were based on a limited number of MyPlate food categories. Finally, the modeling of an optimum breakfast would benefit from formal diet optimization methods such as linear programming.

## 5. Conclusions

The present analyses showed that the American breakfast was already a nutrient dense meal; however, there is room for improvement. While providing 20% of daily energy, breakfast provided higher amounts of key micronutrients. Diet quality of breakfast consumers, assessed using the NRF9.3 score for diets showed that higher diet quality NRF9.3 tertiles were associated with greater consumption of nutrients and food groups of interest.

## Figures and Tables

**Table 1:** Frequency (%) and 95% confidence limits of breakfast consumption by age group and by key demographics.

	Children/Adolescents (*N* = 4057)			Adults/Older Adults (*N* = 10,431)		
	All (*N* = 4057)	Skippers *N* = 761 (18%)	Consumers *N* = 3296 (82%)	All (*N* = 10431)	Skippers *N* = 2162 (19.7%)	Consumers *N* = 8269 (80.3%)
Age (y)						
6–13	2511	12.53 (10.29–14.76)	87.47 (85.24–89.71)			
13–18	1546	25.76 (21.71–29.81)	74.24 (70.19–78.29)			
18–55				6594	23.51 (21.38–25.63)	76.49 (74.37–78.62)
>55				3837	12.5 (10.89–14.11)	87.5 (85.89–89.11)
		<0.001			<0.001	
Gender						
Male	2073	17.68 (15.18–20.18)	82.32 (79.82–84.82)	5092	21.41 (19.78–23.05)	78.59 (76.95–80.22)
Female	1984	18.36 (15.13–21.59)	81.64 (78.41–84.87)	5339	18.04 (15.9–20.18)	81.96 (79.82–84.1)
		0.66			<0.005	
Race/ethnicity						
Non-Hispanic White	1010	16.47 (12.87–20.07)	83.53 (79.93–87.13)	4225	17.43 (15.5–19.35)	82.57 (80.65–84.5)
Non-Hispanic Black	1119	26.21 (21.86–30.57)	73.79 (69.43–78.14)	2443	27.09 (24.44–29.74)	72.91 (70.26–75.56)
Mexican American	854	20.35 (17.17–23.53)	79.65 (76.47–82.83)	1252	24.28 (19.98–28.58)	75.72 (71.42–80.02)
Asian	415	12.87 (7.64–18.11)	87.13 (81.89–92.36)	1199	20.56 (17.18–23.94)	79.44 (76.06–82.82)
Other Hispanic	424	16.46 (13.00–19.92)	83.54 (80.08–87.00)	984	19.61 (15.52–23.7)	80.39 (76.3–84.48)
Other/mixed race	235	10.38 (4.30–16.47)	89.62 (83.53–95.7)	328	26.15 (20.72–31.58)	73.85 (68.42–79.28)
		<0.001			<0.001	
Family IPR ^1,2^						
<1.3	1739	22.97 (19.92–26.01)	77.03 (73.99–80.08)	3445	26.66 (23.83–29.5)	73.34 (70.5–76.17)
1.3–1.849	498	20.92 (15.81–26.03)	79.08 (73.97–84.19)	1176	20.29 (16.71–23.87)	79.71 (76.13–83.29)
1.85–2.99	561	22.79 (15.75–29.84)	77.21 (70.16–84.25)	1515	19.88 (16.74–23.02)	80.12 (76.98–83.26)
≥3.0	996	9.93 (6.70–13.17)	90.07 (86.83–93.3)	3511	15.46 (13.42–17.5)	84.54 (82.5–86.58)
		<0.001			<0.001	
Education ^3^						
<High school				2130	24.58 (21.49–27.67)	75.42 (72.33–78.51)
High school				2149	20.3 (17.59–23.01)	79.7 (76.99–82.41)
Some college				3040	22.08 (19.77–24.39)	77.92 (75.61–80.23)
≥College graduate				2522	12.98 (10.73–15.23)	87.02 (84.77–89.27)
					<0.001	

^1^ IPR stands for Income to poverty ratio; ^2^ In children (resp in adults), 263 (resp. 784) missing IPR were removed from the analysis; ^3^ In adults, 590 missing education information were removed from the analysis.

**Table 2:** Mean (±SE) NRF 9.3 scores for breakfast consumers by age and socio-demographics.

	All (*n* = 11,565)	Children (*n* = 2152)	Adolescents (*n* = 1144)	Adults (*n* = 4955)	Older adults (*n* = 3314)
Total		449.1 (5.82)	407.11 (7.15)	420.28 (4.97)	483.07 (6.12)
		<0.0001			
Gender					
Male	5663	446.67 (6.67)	420.39 (8.66)	403.42 (5.75)	465.62 (6.94)
Female	5902	451.78 (8.38)	394.09 (9.69)	436.43 (6.14)	497.92 (6.89)
		0.5997	0.0362	<0.001	<0.001
Race/ethnicity					
Non-Hispanic White	4346	435.52 (11.15)	390.95 (13.5)	419.53 (7.48)	487.61 (6.79)
Non-Hispanic Black	2664	432.50 (8.57)	380.27 (11.29)	371.94 (5.79)	428.25 (8.89)
Mexican American	1647	486.34 (9.53)	458.23 (8.46)	437.89 (8.33)	477.55 (11.36)
Asian	1303	500.59 (17.69)	469.59 (23.45)	482.85 (7.17)	520.76 (10.7)
Other Hispanic	1164	470.64 (16.56)	413.57 (21.68)	430.59 (7.78)	508.27 (10.67)
Other/mixed race	441	447.68 (26.88)	403.08 (15)	411.78 (20.73)	436.06 (39.03)
		0.0080	<0.001	<0.001	<0.001
Family income-to-poverty ratio ^1^					
<1.3	3912	448.64 (8.98)	403.2 (12.27)	381.05 (8.26)	459.09 (7.56)
1.3–1.849	1310	459.92 (14.72)	416.6 (22.4)	403.76 (14.6)	462.46 (10.98)
1.85–2.99	1683	433.01 (16.92)	365.12 (19.64)	405.24 (10.84)	464.92 (11.12)
≥3.0	3835	448.81 (12.17)	425.61 (11.66)	451.36 (5.96)	500.84 (7.79)
		0.4986	0.0676	<0.001	0.0014
Education ^1^					
<High School	1625			383.05 (9.01)	457.81 (10.14)
High school	1707			367.08 (9.88)	453.53 (11.2)
Some college	2362			407.77 (6.63)	482.68 (9.27)
≥College graduate	2181			482.18 (7.56)	517.95 (9.23)
				<0.001	<0.001
Breakfast consumption	*N* = 14,488				
Eat	11,565	449.10 (5.81)	407.10 (7.15)	420.28 (4.97)	483.07 (6.12)
Skip	2923	341.70 (14.67)	327.18 (12.53)	310.55 (7.63)	388.21 (9.70)
		<0.001	<0.001	<0.001	<0.001

^1^ Missing values were removed from the analysis.

**Table 3:** Amounts of selected food groups consumed at breakfast across tertiles of NRF 9.3 score by age group. Data for breakfast consumers only.

	Children/Adolescents (*N* = 3296)						Adults/Older Adults (*N* = 8269)					
	T1	T2	T3	*p* *	*p* **	*p* ***	T1	T2	T3	*p* *	*p* **	*p* ***
Citrus fruits †	0.02 (0)	0.04 (0.01)	0.07 (0.02)	0.0104	0.015	0.0058	0.02 (0)	0.06 (0.01)	0.1 (0.01)	<0.001	<0.001	<0.001
Juice (cup) †	0.09 (0.01)	0.19 (0.02)	0.21 (0.02)	<0.001	<0.001	<0.001	0.07 (0.01)	0.16 (0.02)	0.17 (0.01)	<0.001	<0.001	<0.001
Other fruits †	0.06 (0.02)	0.09 (0.01)	0.12 (0.01)	0.0325	0.033	0.0343	0.06 (0)	0.13 (0.01)	0.24 (0.01)	<0.001	<0.001	<0.001
Whole grains ‡	0.18 (0.02)	0.3 (0.03)	0.47 (0.03)	<0.001	0.000	<0.001	0.22 (0.02)	0.47 (0.03)	0.7 (0.03)	<0.001	<0.001	<0.001
Refined grains ‡	1.63 (0.06)	1.24 (0.07)	0.81 (0.04)	<0.001	0.000	<0.001	1.54 (0.04)	1.28 (0.04)	0.79 (0.03)	<0.001	<0.001	<0.001
Meat/poultry/fish ‡	0.41 (0.05)	0.29 (0.03)	0.1 (0.01)	<0.001	0.000	<0.001	0.61 (0.03)	0.38 (0.03)	0.16 (0.01)	<0.001	<0.001	<0.001
Eggs ‡	0.30 (0.03)	0.29 (0.03)	0.2 (0.03)	0.0457	0.545	0.5191	0.44 (0.03)	0.44 (0.02)	0.31 (0.03)	0.0023	<0.001	0.0098
Soy, nuts, legumes ‡	0.06 (0.01)	0.05 (0.01)	0.09 (0.02)	0.1455	0.047	0.0738	0.13 (0.02)	0.19 (0.02)	0.31 (0.02)	<0.001	<0.001	<0.001
Milk †	0.41 (0.03)	0.6 (0.03)	0.74 (0.03)	<0.001	0.000	<0.001	0.23 (0.01)	0.35 (0.02)	0.52 (0.01)	<0.001	<0.001	<0.001
Yogurt †	0.01 (0)	0.01 (0)	0.03 (0.01)	0.0312	0.038	0.0439	0.02 (0)	0.03 (0)	0.04 (0.01)	<0.001	<0.001	0.0027
Cheese †	0.07 (0.01)	0.08 (0.01)	0.05 (0.01)	0.0217	0.393	0.3791	0.13 (0.01)	0.1 (0.01)	0.05 (0)	<0.001	<0.001	<0.001

* Unadjusted *p*-value; ** *p*-value adjusted for total energy at breakfast; *** *p*-value adjusted for energy at breakfast, ethnicity, income to poverty ratio, education (adults only) and gender; † Units for citrus fruits, juice, other fruits, milk, yogurt and cheese are cup-equivalents; ‡ Units for whole grains, refined grains, meat/poultry/fish (MPF), eggs, soy, nuts and legumes are ounce-equivalents.

**Table 4:** Mean (standard error) intake of nutrients at breakfast (among consumers of breakfast only) across tertiles of NRF9.3 score by age group.

	Children						Adults					
	T1	T2	T3	*p* *	*p* **	*p* ***	T1	T2	T3	*p* *	*p* **	*p* ***
Ranges of NRF	[−568, 378]	[378, 506]	[506, 866]				[−822, 376]	[376, 521]	[521, 878]			
NRF9.3	258 (5)	443 (2)	595 (3)	<0.001	<0.001	<0.001	255 (2)	450 (1)	622 (2)	0.0000	<0.001	<0.001
**Vitamins/minerals in NRF9.3 model**												
Vitamin A, RAE (mcg)	184 (9)	242 (9)	294 (8)	<0.001	<0.001	<0.001	158 (8)	212 (7)	306 (15)	<0.001	<0.001	<0.001
Vitamin C (mg)	13 (1)	24 (2)	28 (2)	<0.001	<0.001	<0.001	13 (1)	24 (2)	35 (2)	<0.001	<0.001	<0.001
Vitamin D (mcg)	2 (0.1)	3 (0.1)	3 (0.1)	<0.001	<0.001	<0.001	2 (0.1)	2 (0.1)	3 (0.1)	<0.001	<0.001	<0.001
Calcium (mg)	242 (10)	312 (12)	372 (9)	<0.001	<0.001	<0.001	212 (5)	268 (7)	348 (6)	<0.001	<0.001	<0.001
Iron (mg)	4 (0.2)	5 (0.2)	6.3 (0.3)	<0.001	<0.001	<0.001	4 (0.2)	5 (0.1)	7 (0.2)	<0.001	<0.001	<0.001
Potassium (mg)	415 (14)	529 (18)	581 (12)	<0.001	<0.001	<0.001	540 (10)	640 (13)	789 (15)	<0.001	<0.001	<0.001
Magnesium (mg)	44 (1)	53 (1)	64 (2)	<0.001	<0.001	<0.001	57 (1)	70 (1)	95 (2)	<0.001	<0.001	<0.001
Sodium (mg)	656 (24)	572 (23)	455 (15)	<0.001	<0.001	<0.001	784 (20)	677 (22)	507 (10)	<0.001	<0.001	<0.001
**Vitamins/minerals not in the NRF9.3 model**												
Retinol (mcg)	180 (10)	235 (9)	281 (7)	<0.001	<0.001	<0.001	150 (8)	196 (7)	264 (6)	<0.001	<0.001	<0.001
Thiamin (mg)	0.4 (0.01)	0.5 (0.02)	0.6 (0.02)	<0.001	<0.001	<0.001	0.4 (0.01)	0.5 (0.01)	0.6 (0.01)	<0.001	<0.001	<0.001
Riboflavin (mg)	0.6 (0.02)	0.7 (0.02)	0.9 (0.02)	<0.001	<0.001	<0.001	0.7 (0.02)	0.8 (0.01)	0.9 (0.01)	<0.001	<0.001	<0.001
Niacin (mg)	5 (0.2)	6 (0.2)	6 (0.2)	<0.001	<0.001	<0.001	6 (0.2)	6 (0.1)	7 (0.2)	0.0010	<0.001	<0.001
Vitamin B6 (mg)	0.4 (0.02)	0.6 (0.03)	0.7 (0.03)	<0.001	<0.001	<0.001	0.5 (0.02)	0.6 (0.02)	0.8 (0.02)	<0.001	<0.001	<0.001
Vitamin B12 (mcg)	1 (0.1)	2 (0.1)	2 (0.1)	<0.001	<0.001	<0.001	1 (0.1)	2 (0.1)	2 (0.1)	<0.001	<0.001	<0.001
Vitamin E (mg)	1 (0.1)	1 (0.1)	1 (0.1)	0.4962	0.139	0.1650	2 (0.1)	2 (0.1)	3 (0.2)	<0.001	<0.001	<0.001
Folate, DFE (mcg)	159 (9)	195 (10)	258 (12)	<0.001	<0.001	<0.001	135 (6)	181 (5)	265 (10)	<0.001	<0.001	<0.001
Total folates (mcg)	107 (6)	130 (6)	167 (7)	<0.001	<0.001	<0.001	96 (4)	128 (3)	180 (6)	<0.001	<0.001	<0.001
Phosphorus (mg)	301 (11)	329 (12)	352 (6)	0.0005	<0.001	<0.001	318 (7)	339 (7)	381 (8)	<0.001	<0.001	<0.001
Zinc (mg)	2 (0.1)	3 (0.1)	4 (0.1)	<0.001	<0.001	<0.001	2 (0.1)	3 (0.07)	4 (0.1)	<0.001	<0.001	<0.001
Copper (mg)	0.2 (0)	0.2 (0.01)	0.2 (0.01)	<0.001	<0.001	<0.001	0.2 (0.01)	0.3 (0.01)	0.3 (0.01)	<0.001	<0.001	<0.001
Selenium (mcg)	22 (0.8)	21 (0.94)	18 (0.7)	<0.001	0.573	0.8552	27 (0.6)	26 (0.8)	24 (0.9)	0.0316	<0.001	<0.001

* *p*-Value unadjusted; ** *p*-value adjusted for energy at breakfast; *** *p*-value adjusted for energy at breakfast, ethnicity, income to poverty ratio, education (adults only) and gender.
